# Interconnected worlds: a comprehensive review of fungal defenses, antimicrobial resistance, and their evolutionary dynamics

**DOI:** 10.3897/imafungus.17.171995

**Published:** 2026-01-27

**Authors:** Yuan Sui, Mir Muhammad Nizamani, Entaj Tarafder, Hai-Li Zhang, Qian Zhang, Krishnendu Acharya, Jit Sarkar, Ghulam Muhae-Ud-Din, Yong Wang

**Affiliations:** 1 Chongqing Key Laboratory for Germplasm Innovation of Special Aromatic Spice Plants, College of Smart Agriculture/Institute of Special Plants, Chongqing University of Arts and Sciences, Yongchuan, Chongqing 402160, China Centre of Advanced Study, University of Calcutta Kolkata India; 2 Department of Plant Pathology, College of Agriculture, Guizhou University, Guiyang 550025, China Institute of Special Plants, Chongqing University of Arts and Sciences Chongqing China; 3 Sanya Nanfan Research Institute of Hainan University, Hainan Yazhou Bay Seed Laboratory, Hainan University, Sanya 572025, China College of Agriculture, Guizhou University Guiyang China; 4 Molecular and Applied Mycology and Plant Pathology Laboratory, Department of Botany, Centre of Advanced Study, University of Calcutta, Kolkata 700019, India Hainan Yazhou Bay Seed Laboratory, Hainan University Sanya China

**Keywords:** Adaptive responses, cell wall integrity, efflux pumps, enzymatic breakdown, horizontal gene transfer, interdisciplinary collaboration

## Abstract

Fungal defense mechanisms and antimicrobial resistance to therapeutic remedies represent a complex and evolving challenge. This review explores the multifaceted processes that determine fungal resistance and covers cellular, evolutionary, and global aspects. Key factors, such as cell wall integrity, efflux pumps, and adaptive responses, are examined, along with interdisciplinary analytical techniques used to elucidate defense mechanisms. Evolutionary drivers, including natural selection and horizontal gene transfer, are also discussed. The review emphasizes the importance of global coordination, personalized medicine, ethical principles, and sustainable practices in both healthcare and agriculture to address the growing problem of antimicrobial resistance to therapeutic drugs. It synthesizes existing literature and offers recommendations for future research and initiatives designed to support a global effort capable of proactively addressing antimicrobial resistance and overcoming fungal defense mechanisms, thereby mitigating their impact on human health and food production.

## Introduction

The growing challenge of antimicrobial resistance (AMR) to therapeutic drugs represents a critical intersection of clinical medicine, agricultural practices, and environmental health. Resistance mechanisms in human pathogens, plant pathogens, and native fungi are interconnected and demonstrate remarkable parallels across ecosystems. This review presents a central thesis framed within the integrative One Health paradigm, which recognizes the interdependence of human, animal, plant, and environmental health: the evolutionary dynamics of fungal defense mechanisms operate across ecosystem boundaries, with AMR in one domain directly influencing resistance landscapes in others. For example, the agricultural use of azole fungicides has been directly linked to the emergence of clinically resistant *Aspergillus fumigatus* strains, illustrating how human activities create cross-domain evolutionary pressure (Bastos et al. 2021; Fisher et al. 2022). This cross-domain connectivity underscores the value of an integrated perspective when studying the evolution of fungal defense and resistance. Throughout this review, we explicitly trace these linkages, demonstrating, for instance, how horizontal gene transfer (HGT) events in plant pathogens parallel those in clinical fungi and how resistance mechanisms in agriculture inform clinical therapeutic failures.

To provide this synthesis, we establish a key conceptual distinction: fungal defense mechanisms encompass the broad adaptive traits that protect fungi from environmental stressors, host immunity, and microbial competition. AMR, in contrast, refers specifically to the ability to tolerate, mitigate, or neutralize therapeutic antifungal agents. While much AMR is built upon components of a pre-existing defensive repertoire, not all defense mechanisms confer therapeutic resistance. This framework allows the tracing of how basal fungal defenses are co-opted and refined under drug selection to become stable, heritable AMR.

Despite many academic and clinical advances, the integration of knowledge across clinical, agricultural, and environmental contexts remains insufficient. This review seeks to fill this critical gap by offering a comprehensive synthesis of how fungal resistance mechanisms evolve across different domains and impact each other. Our understanding of fungal biology has evolved through advances in technology. Initial morphological studies have progressed to recent genomic approaches that have revealed the remarkable plasticity of fungal genomes (Latgé 2007; Taylor et al. 2015). However, research has largely remained within distinct disciplines, with limited integration of clinical, agricultural, and environmental studies. This review aims to provide a novel synthesis by examining fungal defense mechanisms not as isolated processes but as an integrated system that operates across biological scales and ecological contexts. We discuss how cellular defenses, such as cell wall integrity and efflux capacity, serve as the foundation for resistance evolution, and how evolutionary processes, such as HGT and hybridization, facilitate the rapid spread of resistance traits across fungal populations and species. We also examine how anthropogenic activities—particularly the use of antimicrobials in medicine and agriculture—have accelerated these evolutionary processes.

A holistic understanding of the evolution of defense mechanisms and resistance is crucial for the development of effective countermeasures.

**Figure 1.**
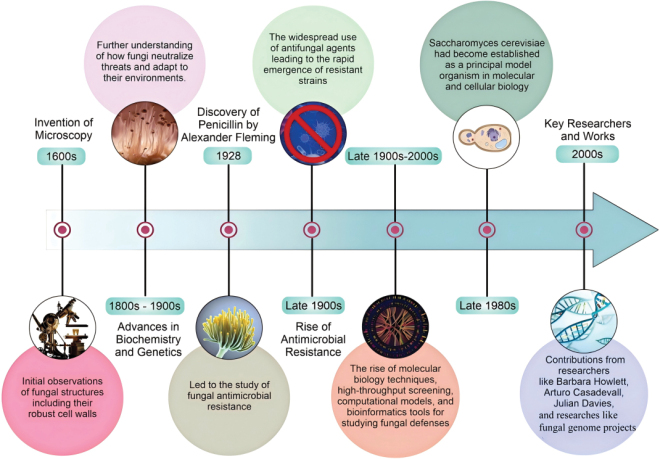
Milestones in fungal defense research. This timeline highlights key technological and conceptual advances, from early morphological studies to modern genomics and systems biology, that have shaped our understanding of fungal resistance mechanisms.

Milestones in fungal defense research are summarized (Fig. 1). In agriculture, fungal defense mechanisms undermine the efficacy of both conventional fungicides and biological control agents, thereby threatening global food security (Dean et al. 2012). In clinical settings, AMR renders classical antifungal drugs increasingly ineffective, especially in immunocompromised patients (Fisher et al. 2018). Paradoxically, fungi also serve as a rich source of antimicrobial compounds, highlighting the complex evolutionary arms race that shapes microbial communities (Keller et al. 2005). The persistent capacity of fungal pathogens to adapt and evolve calls for a shift from reactive to proactive strategies. Current approaches largely address resistance after it emerges rather than anticipating the evolution of adaptive responses. Overcoming these limitations will require the integration of knowledge across disciplines, from molecular biology to microbial and ecosystem ecology, to develop sustainable management strategies that account for the interconnected development of microbial resistance in humans, animals, plants, and the environment.

The primary objective of this review is to provide a broader, holistic understanding of antifungal resistance based on the premise that fungal defense mechanisms are not merely a collection of traits but represent an interconnected, evolving network that both impacts and is impacted across clinical, agricultural, and environmental boundaries. This review is structured to systematically connect molecular mechanisms to their evolutionary and real-world consequences. Specifically, we aim to:

Present and discuss the core arsenal of fungal defense mechanisms, from physical barriers (e.g., cell wall integrity) to dynamic molecular and genetic responses (e.g., efflux pumps, enzymatic degradation, and adaptive regulatory networks). This overview is the focus of Section 3.

Examine how defense mechanisms are co-opted and amplified into clinical and agricultural resistance through evolutionary processes. In this context, Section 4 critically examines the roles of HGT, hybridization, and anthropogenic selection in transforming basal defense mechanisms into robust, stable, and widespread resistance.

Identify critical translational gaps and propose a shift toward interdisciplinary solutions. In Sections 5, 6, and 7, we highlight the limitations of current management strategies and argue that overcoming the adaptive resilience of fungal pathogens necessitates an approach that integrates insights from medicine, agriculture, environmental science, and governmental regulatory policy. This review frames the problem through a perspective of interconnectedness and evolution, based on the premise that the survival of fungal pathogens is a direct function of their ability to integrate defense, adaptation, and resistance. We conclude that addressing this escalating problem will require an interdisciplinary approach and that success is fundamentally dependent on a more holistic strategy.

## Background

### Resistance to antimicrobial agents

Resistance to antifungal agents is an escalating global challenge, with several clinically important fungi showing reduced susceptibility across multiple drug classes. *Nakaseomyces glabratus* (formerly *Candida glabrata*) commonly exhibits decreased azole susceptibility, particularly to fluconazole, and resistance to echinocandins has been associated with mutations in *fks* genes (Pfaller et al. 2013). *Candidozyma auris* (formerly *Candida auris*) is an emerging multidrug-resistant pathogen; some isolates display resistance to multiple antifungal classes, substantially limiting therapeutic options (Argomedo et al. 2020). *A. fumigatus* has also developed clinically significant resistance to triazoles, frequently linked to mutations in *cyp51A*, with resistant strains reported in both clinical and natural settings (Bastos et al. 2021; White et al. 2021). In addition, *Cryptococcus neoformans* may develop fluconazole resistance during prolonged therapy, complicating the management of cryptococcal meningitis (Castanheira et al. 2016).

At the molecular level, antifungal resistance arises through multiple mechanisms. These include increased drug efflux mediated by ATP-binding cassette (ABC) and major facilitator superfamily (MFS) transporters—well documented in *Candida albicans* and *N. glabratus*—which reduce intracellular drug accumulation (Morschhäuser 2010). Genomic plasticity, including aneuploidy and gene amplification, such as amplification of *ERG11*, further contributes to elevated resistance in *Candida* spp. (Handelman and Osherov 2022). Additional mechanisms include drug-target alterations, biofilm-associated tolerance, and HGT (Perfect 2017; Fisher et al. 2018). From a broader perspective, antifungal resistance is promoted by selective pressure arising from antifungal exposure, including inappropriate or excessive use and suboptimal disease management practices (Pfaller and Diekema 2010). Strengthened surveillance, improved antifungal stewardship, and the continued development of new therapeutic strategies are therefore essential, as rising resistance is associated with increased treatment failure, healthcare costs, and mortality (Brown et al. 2012; Perlin 2014; Fisher et al. 2018).

### Analytical methods used in the study of fungal defense mechanisms

Studies of fungal defense mechanisms increasingly integrate classical microbiology with modern molecular and computational approaches. Foundational methods, including culturing, phenotyping, and nutrient requirement analyses, remain central to identifying resistant strains and documenting observable growth and morphological responses under controlled conditions (Aleklett et al. 2021; Rämä and Quandt 2021). Substantial advances in understanding resistance have emerged from genomics, transcriptomics, proteomics, and metabolomics, which elucidate the genetic and biochemical bases of defense, including pathway-level responses associated with antifungal exposure (Naranjo-Ortiz and Gabaldón 2019; Ball et al. 2020; Crandall et al. 2020). Omics approaches have clarified key resistance mechanisms, such as efflux pump regulation, lipid remodeling, and biofilm formation, in clinically relevant fungi, including *C. auris* (Zamith-Miranda et al. 2019). High-throughput screening has further accelerated antifungal discovery by enabling rapid testing of large compound libraries and identifying how candidate agents disrupt resistance-associated pathways, although findings from simplified *in vitro* systems may not fully reflect biological complexity (Sun et al. 2021; Nizamani et al. 2023).

To interpret these data-rich outputs, bioinformatics and systems biology have become indispensable for integrating multi-omic datasets, detecting functional interactions, and tracing evolutionary patterns in fungal populations (Culibrk et al. 2016). Tools such as MATLAB support systems-level modeling of regulatory and metabolic networks, while MEGA enables comparative sequence analyses and phylogenetic reconstruction to infer the origins and spread of resistance-associated genes (Kumar et al. 2008; Tamura et al. 2011; Xiao et al. 2017). Population genomic tools, including POPBAM, extend these analyses by estimating nucleotide diversity and population divergence from short-read alignments (Garrigan 2013). Integrative platforms such as EvoPipes.net further connect next-generation sequencing outputs with ecological and evolutionary variables to examine adaptation in a broader context (Prins et al. 2012). Despite their strengths, computational approaches remain sensitive to data quality and model assumptions, and omics and screening studies require careful validation to avoid overinterpretation of correlative patterns. Consequently, *in vivo* models and ecological investigations remain essential complementary approaches that anchor molecular and computational findings in realistic host and environmental contexts, despite their higher resource demands and variability across experimental settings.

### Host–pathogen interactions and innate immunity

Research on host–pathogen interactions has substantially advanced understanding of fungal defense mechanisms and host immunity. Studies of cell-mediated immunity demonstrate that macrophages and neutrophils play central roles in controlling fungal infections (Casadevall 1995). Investigations of cytokine responses further show how modulation of immune signaling can enhance antifungal defense responses, particularly in immunocompromised patients (Antachopoulos and Roilides 2005). Genomic initiatives, such as the *Candida* Genome Sequencing Project, have identified numerous antifungal resistance genes and metabolic pathways, providing valuable targets for both agricultural biocontrol strategies and clinical therapeutic development (Davies and Davies 2010). Collectively, immunological and genetic research continues to advance the development of novel strategies to combat fungal pathogens.

### The spectrum of antifungal resistance: intrinsic vs. acquired

Intrinsic resistance refers to the innate ability of a fungal species to resist specific antifungal agents. This resistance is genetically encoded and is not induced by prior exposure to antifungal compounds. For example, *Pichia kudriavzevii* (formerly *Candida krusei*) is innately resistant to fluconazole, whereas *Aspergillus terreus* is naturally resistant to amphotericin B due to differences in cellular targets and membrane composition (Billé 2000; Nguyen et al. 2024). These innate traits are stable and evolutionarily conserved, reflecting adaptive mechanisms that evolved prior to the modern use of antifungal agents (Edlind 2007).

In contrast, acquired resistance develops in previously susceptible fungal strains through genetic selection driven by exposure to antifungal compounds. This process involves mutations in drug targets (e.g., *ERG11* in *C. albicans*), overexpression of efflux pumps, biofilm formation, and chromosomal rearrangements (Mallick et al. in press). For example, prolonged fluconazole exposure has been associated with the emergence of azole-resistant *C. glabrata* and *A. fumigatus* (Drew and Townsend 2010; Jensen et al. 2016).

From a clinical standpoint, intrinsic resistance underscores the importance of accurate pathogen identification and appropriate selection of antifungal therapeutics prior to treatment. In contrast, acquired resistance is often detected during therapy, particularly when drug exposure is prolonged or suboptimal, resulting in treatment failure (Sanguinetti et al. 2015). Comparatively, intrinsic resistance reflects long-standing evolutionary, ecological, and biological adaptation, whereas acquired resistance represents an adaptive response to antifungal selection pressure present in both clinical and environmental settings (Edlind 2007). Consistent with these concepts, class-specific antifungal targets and dominant resistance pathways are listed (Table 1).

**Table 1.** Major antifungal drug classes: representative agents, primary targets, key pathogens, and common resistance mechanisms.

Class	Representative drugs	Primary target / mechanism of action	Main pathogens targeted	Dominant resistance mechanisms	Supporting references
Azoles	Fluconazole, Itraconazole, Voriconazole, Posaconazole	Inhibit *lanosterol 14α-demethylase (CYP51A)*, blocking ergosterol synthesis	*Candida* spp., *Aspergillus fumigatus*	Point mutations in *CYP51A* (e.g., TR34/L98H), efflux pump overexpression (*CDR1*, *MDR1*), biofilm protection, and aneuploidy-mediated tolerance	(Osset-Trénor et al. 2023; Vitiello et al. 2023)
Echinocandins	Caspofungin, Micafungin, Anidulafungin	Inhibit *β-1,3-glucan synthase* (Fks1p/Fks2p), disrupting cell wall biosynthesis	*Candida* spp., *Aspergillus* spp. (limited)	Mutations in *FKS1/FKS2* “hotspot” regions, compensatory upregulation of chitin synthesis	(Prasad et al. 2016; Perlin et al. 2017)
Polyenes	Amphotericin B, Nystatin	Bind directly to *ergosterol* in fungal membranes, forming pores and causing ion leakage	Broad-spectrum (*Candida*, *Cryptococcus*, *Aspergillus*)	Altered sterol composition (loss of ergosterol), oxidative stress adaptation	(Prasad et al. 2016)
Pyrimidine analogues	Flucytosine (5-FC)	Converted to 5-fluorouracil, inhibiting RNA and DNA synthesis	*Candida* spp., *Cryptococcus neoformans*	Mutations in *FCY1*, *FCY2*, or *FUR1* disrupt drug uptake or metabolism	(Vitiello et al. 2023)
Allylamines	Terbinafine, Naftifine	Inhibit *squalene epoxidase*, a key enzyme in ergosterol biosynthesis	Dermatophytes (*Trichophyton*, *Microsporum*)	Mutations in *SQLE* gene, efflux pump overexpression	(Nigam, 2015)
Novel / experimental agents	Olorofim, Fosmanogepix, Ibrexafungerp	Inhibit *dihydroorotate dehydrogenase (DHODH)*, *GPI-anchor synthesis*, or *glucan synthase*	Emerging *Candida* and *Aspergillus* spp.	Resistance under investigation; early evidence suggests target-site adaptation and stress response modulation	(Dominguez et al. 2023)

## Factors contributing to resistance

### Cell wall integrity

Fungal cell walls are primarily composed of chitin, glucans, and proteins, which play a crucial role in maintaining cell integrity and mediating host–pathogen interactions. The composition of fungal cell walls is unique to fungi and differs significantly from that of animals and plants, making cell wall components prime targets for antifungal drug development (Lima et al. 2019; Alcazar-Fuoli et al. 2020). The dynamic structure of the fungal cell wall presents numerous opportunities for pharmacological intervention (Bowman and Free 2006). Intrinsic resistance to antifungal agents in fungi is often attributable to specific cell wall features. For example, the low β-glucan content in the cell walls of fungi belonging to the *Mucorales* renders them naturally resistant to echinocandin drugs. In parallel, chitin and β-glucans are recognized by the host immune system through pattern recognition receptors, triggering complex interactions related to pathogen detection and immune evasion (Alcazar-Fuoli et al. 2020). Consequently, reduced β-glucan content decreases the likelihood of host immune recognition.

The cell walls of *C. neoformans* provide an example of fungal adaptation in which environmental factors influence cell wall composition, thereby affecting host–pathogen dynamics (Garcia-Rubio et al. 2020; Upadhya et al. 2023). Cell wall modifications in resistant strains further contribute to the development of acquired antifungal resistance (Lima et al. 2019). Cell wall composition varies widely across species and strains, including *Candida*, *Cryptococcus*, and *Aspergillus*, highlighting the need for species-specific studies (Gow and Lenardon 2023). Novel therapeutics, such as ibrexafungerp, target β-(1,3)-glucan synthesis and have demonstrated broad efficacy without the cross-resistance issues observed with echinocandins (McLellan et al. 2012; Ibe and Munro 2021).

### Efflux pumps

Resistance to antifungal agents often involves alterations in the activity of efflux pumps, which are membrane transporter proteins that actively expel toxic compounds from the cell. This process decreases intracellular drug concentrations, thereby reducing treatment efficacy (Osset-Trénor et al. 2023). A critical distinction must be made between intrinsic resistance, mediated by the constitutive basal expression of these pumps in certain species, and acquired resistance, which arises through their upregulation or mutation in direct response to antifungal drug pressure. The functional complexity of efflux pumps—primarily ABC and MFS transporters—reflects the diverse evolutionary strategies fungi employ to counteract antimicrobial agents. Notably, some natural compounds can inhibit efflux pump function and thereby restore the efficacy of standard therapies. For example, in *C. albicans* strains with acquired resistance, the compound vanillin suppresses azole-induced overexpression of the *CDR2* gene and alters the cellular localization of its product, the ABC transporter CaCdr2p. Similarly, the natural compound myriocin enhances fluconazole efficacy by interfering with the membrane localization of the Cdr1 pump without affecting expression of the gene encoding this transporter (Yang et al. 2021; Wang et al. 2022). In addition, a novel light-triggered nanoplatform has been shown to reverse acquired efflux pump activity while modulating heat shock protein expression, offering a promising chemo-photothermal strategy for combating resistant fungi (Yang et al. 2021).

Efflux pump activity is subject to complex genetic and post-transcriptional regulation, which facilitates rapid adaptation. For example, antifungal agent–induced alternative splicing in *Trichophyton rubrum* generates altered efflux pump transcripts that directly contribute to adaptive, acquired resistance (Lopes et al. 2022). A detailed understanding of efflux pump structure, regulation, and kinetics is therefore essential for the development of effective inhibitors. Combining conventional antifungal agents with efflux pump inhibitors represents a promising therapeutic strategy for overcoming acquired resistance and improving clinical outcomes (Jayan and Gupta 2023).

### Enzymatic degradation

Fungi secrete extracellular enzymes, such as aspartic proteases, that function primarily as virulence factors by facilitating nutrient acquisition, host tissue invasion, and evasion of immune recognition. Although these enzymes form part of the fungal defense arsenal against host innate immunity, their role in direct antifungal drug resistance is often indirect. For example, secreted aspartic proteases (Saps) in *Candida* spp. can degrade host antimicrobial peptides, thereby promoting survival in hostile host environments. In some cases, overexpression of *SAP* genes has been correlated with reduced azole susceptibility, suggesting that enhanced virulence pathways may secondarily support survival under drug pressure. However, Sap activity does not represent a direct resistance mechanism comparable to target-site mutation or efflux pump overexpression. While the production of these enzymes reflects an intrinsic virulence trait, their contribution to resistance is often linked to adaptive, acquired mechanisms, such as transcriptional upregulation. For instance, hydrolytic enzymes, particularly aspartic proteases, in *Candida* species facilitate tissue invasion, nutrient uptake, and degradation of host defense proteins, making them key virulence determinants as well as potential drug targets (Staniszewska et al. 2017). Saps also inactivate host immune effectors, including antimicrobial peptides (e.g., LL-37), and reduce neutrophil recruitment. This proteolysis-based evasion of host immune responses allows fungal pathogens to escape recognition and persist within host tissues (Rapala-Kozik et al. 2015).

Sap activity further supports biofilm formation, which is a central feature of both intrinsic and acquired resistance in fungi. Biofilms act as physical barriers that shield fungal cells from antifungal drugs and host immune recognition (Bras et al. 2024). Inhibition of Sap enzymes has been shown to reduce biofilm stability and enhance antifungal efficacy, underscoring the importance of these enzymes in resistance-associated phenotypes (Lohse et al. 2020). Moreover, acquired overexpression of specific *SAP* genes has been associated with increased azole resistance (El-Houssaini et al. 2019).

Extracellular proteases secreted by species of *Candida*, *Aspergillus*, *Penicillium*, and *Cryptococcus* disrupt host barriers, promote colonization, and facilitate infection (Satala et al. 2023). Aspartic proteases also contribute to cell wall maintenance, biofilm development, immune evasion, and modulation of inflammatory responses, highlighting their broad role in fungal pathogenicity (Zawrotniak et al. 2023). Collectively, these findings establish the significant role of proteolytic enzymes in fungal virulence and their contribution to acquired resistance, supporting their potential as targets for antifungal therapeutic development.

### Adaptive responses

Fungi possess a remarkable adaptive capacity that enables survival in unfavorable and fluctuating environments. These adaptive responses rely on complex regulatory networks, including signal transduction pathways and transcriptional regulators, which sense stress and initiate appropriate responses. These networks control gene expression, metabolism, and processes such as biofilm formation, morphology, and the induction of acquired resistance (Fortwendel 2012). In this context, the Ras protein family plays a central role in the growth, differentiation, and virulence of major fungal pathogens. Transcription factors such as *Yap1* and *Skn7* coordinate oxidative stress responses in various fungal species by regulating the expression of suites of stress-related genes (Pais et al. 2016). The Group III histidine kinase AaHSK1 and the yeast HOG1 ortholog AaHOG1 in *Alternaria alternata* function synergistically in fungal signaling pathways (Lin and Chung 2010).

Transcriptional regulators exhibit broad but often species-specific roles. For example, the transcription factor *Crz1* in *Fusarium graminearum* regulates development, secondary metabolism, virulence, and stress responses (Chen et al. 2019). The bZIP transcription factor *MoAP1* controls oxidative stress responses and pathogenicity in *Magnaporthe oryzae* (Guo et al. 2011). Transcription factors in *C. albicans* have been shown to mediate acquired drug resistance and virulence in response to antifungal agents (Vandeputte et al. 2012), whereas *Msn2* and *Msn4* in *Saccharomyces cerevisiae* regulate stress responses and morphology, highlighting the species-specific dynamics of transcriptional regulation (Nicholls et al. 2004). Genomic alterations, such as ploidy changes and mitotic recombination, can further enhance resistance and virulence (Wertheimer et al. 2016; Gusa and Jinks-Robertson 2019). Fungi can also exhibit anticipatory stress responses, pre-activating defense mechanisms that promote immune evasion (Pradhan et al. 2021). This dynamic regulation of stress pathways is a fundamental feature of acquired resistance and contrasts with static, innate traits.

The interconnected nature of fungal adaptive responses, including oxidative stress, biofilm formation, and heat shock responses, is summarized in Suppl. material 1: fig. S1. This model illustrates how key transcription factors (e.g., *Yap1*, *Hsf1*) integrate signals from environmental stressors (e.g., reactive oxygen species, temperature shifts, and antifungal agent exposure) to coordinate unified survival strategies. These processes are regulated by transcription factors such as *Yap1*, *Skn7*, *Crz1*, *Hsf1*, and *Rim101*, whose expression is induced by environmental cues. Given their central roles in fungal adaptation, survival, and pathogenicity, transcriptional regulators represent promising targets for therapeutic intervention in fungal infections.

### Virulence factors versus resistance mechanisms

Virulence factors are genetically encoded traits that enable fungi to infect hosts, evade immune responses, and cause disease. These traits include secretion of tissue-degrading enzymes, toxin production, morphology switching (e.g., yeast-to-hyphae transition), and immune evasion strategies such as capsule formation and melanin synthesis. For example, *C. albicans* uses hyphal morphogenesis and secreted aspartic proteases to invade host tissues, whereas *C. neoformans* employs a polysaccharide capsule to avoid phagocytosis (Karkowska-Kuleta et al. 2009; Day et al. 2018). Resistance, by contrast, refers to the ability of fungi to survive exposure to antifungal agents through specific molecular mechanisms, including target-site mutations, upregulation of efflux pumps, and enzymatic drug degradation or modification. Genomic studies indicate that mutations in key resistance genes (e.g., *ERG11*, *FKS1*) and overexpression of efflux transporters such as *CDR1* in *Candida* species are major determinants of antifungal resistance (Schikora-Tamarit and Gabaldón 2022).

Secreted aspartic proteases (SAPs) comprise a family of enzymes that enable *C. albicans* to degrade host tissue barriers, inactivate immune effector molecules, and facilitate adhesion and invasion of epithelial cells. Studies indicate that most clinical isolates of *C. albicans* express multiple *SAP* genes, particularly *SAP1–SAP6*, and that their expression correlates with tissue invasion and infection severity. Notably, *SAP4–SAP6* are strongly associated with hyphal formation and deep tissue invasion (Ali et al. 2018). Antifungal resistance in *C. albicans* is frequently mediated by the *CDR1* gene, which encodes an ABC efflux transporter. Overexpression of *CDR1* actively exports azole drugs (e.g., fluconazole), reducing intracellular drug concentrations and promoting fungal survival. This mechanism has been well documented in both clinical isolates and experimental models (Lyons and White 2000; Basso et al. 2010; Maras et al. 2021).

Although virulence and resistance are conceptually distinct, they evolve under overlapping selective pressures, including immune defenses, antifungal exposure, and environmental stress. For instance, *C. albicans* and *C. neoformans* exhibit adaptive stress responses in which enhanced thermotolerance and oxidative stress tolerance also promote antifungal resistance (Ball et al. 2024; Gutierrez-Gongora et al. 2024). Biofilms exemplify traits that serve both virulence and resistance functions by promoting adherence to host tissues and immune evasion while simultaneously limiting antifungal penetration. This dual role has been demonstrated in *C. albicans* and *C. auris*, where biofilm formation is correlated with both pathogenic potential and antifungal tolerance (Kadry et al. 2018; Pang et al. 2024; Valente et al. 2025). Genomic innovations mediated by HGT and transposon activity can simultaneously disseminate genes associated with virulence and antifungal resistance. In *A. fumigatus*, large transposons known as “Starships” mobilize gene clusters encoding biofilm-associated virulence factors and antifungal tolerance, generating strain-level heterogeneity (Gluck-Thaler et al. 2025). Reviews of HGT in eukaryotic microbial pathogens indicate that transferred gene suites frequently include determinants of pathogenicity and resistance, accelerating the emergence of highly adaptable pathogens (Gluck-Thaler and Slot 2015). Central molecular regulators, such as the chaperone Hsp90 and its co-chaperones (e.g., Sgt1, Wos2), further illustrate this overlap by stabilizing signaling proteins that govern morphogenesis, stress tolerance, and antifungal resistance (Shapiro et al. 2012; Alaalm et al. 2021).

Understanding the difference between virulence (the ability to cause disease) and resistance (the ability to survive antifungal treatment) is crucial for designing therapies that not only kill fungi but also reduce their capacity to cause harm. For instance, anti-virulence therapies, such as those targeting *C. albicans* secreted proteases or *C. neoformans* capsule formation, can suppress infection severity and immune evasion without fostering selective pressure for resistance development (Gutierrez-Gongora et al. 2024). Antifungal-resistant pathogens such as *C. auris* and *A. fumigatus* exhibit strong virulence traits (e.g., thermotolerance, stress resilience, and biofilm formation) that allow them to persist despite drug exposure. The calcineurin pathway, for example, governs both antifungal resistance and virulence in *C. auris*, highlighting how therapies must address both traits to be successful (Cha et al. 2025). Agricultural azole fungicides used to protect crops share molecular targets with clinical antifungals, leading to cross-resistance in *A. fumigatus* and other “trans-kingdom” pathogens capable of infecting both plants and humans (Pintye et al. 2024). This overlap illustrates the need for an integrated One Health framework that addresses virulence and resistance across medical, veterinary, and agricultural domains (Woods et al. 2023). Effective and sustainable management must combine anti-virulence therapies, resistance-mitigating drugs, and ecological stewardship. For example, combining biological control agents with fungicides in agriculture reduces pathogen virulence and limits resistance evolution (Ons et al. 2020). Similarly, the use of therapeutic strategies in a clinical setting that integrate virulence-suppressing agents with antifungal drugs can enhance complete removal of a pathogen while minimizing selective pressure (Ball et al. 2020).

## Evolution of fungal defense

**Figure 2.**
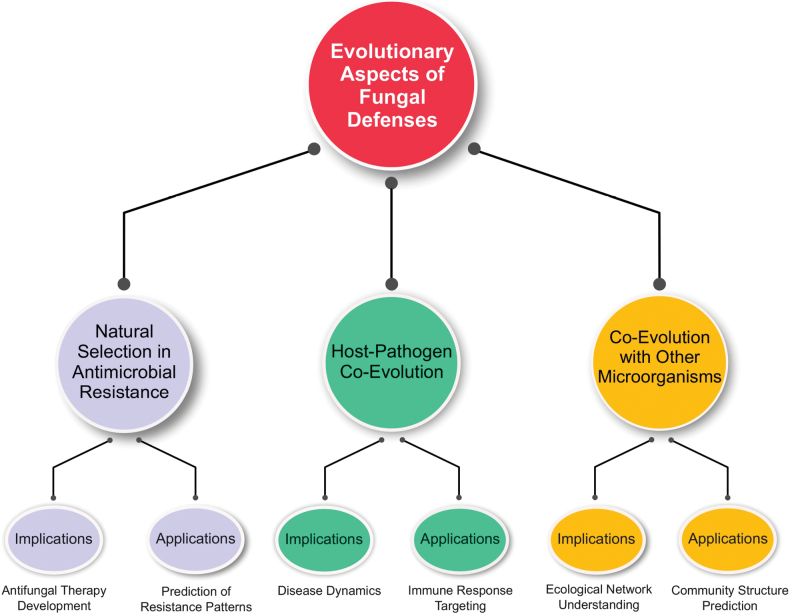
Factors influencing the development of fungal defense strategies and their possible effects.

Fig. 2 presents a conceptual framework for the evolution of fungal defense, highlighting three interconnected drivers: (1) natural selection arising from environmental and ecological pressures, (2) host–pathogen co-evolution that fosters immune evasion, and (3) co-evolution with other microorganisms, which facilitates and enables HGT. This framework underscores how these forces collectively shape both innate and acquired resistance. It highlights three key evolutionary aspects—natural selection, host–pathogen co-evolution, and co-evolution with other microorganisms—that together drive the complex evolutionary processes shaping fungal defense mechanisms, including the evolution of innate resistance and the contemporary emergence of acquired resistance. The framework emphasizes how resistance evolution to antimicrobials, particularly the rapid acquisition of new resistance traits, influences antifungal therapeutic development and aids in predicting resistance patterns and potential treatment strategies. It also highlights the dynamic relationship between fungi and their hosts, especially immune targeting and disease dynamics, as well as the impact of co-evolution between fungi and other microorganisms. Co-evolution plays a critical role in shaping ecological networks and predicting shifts in community structure, including those driven by HGT that can rapidly confer acquired resistance. Collectively, these three categories illustrate the interplay that influences both our understanding of microbial community interactions involving fungi and the development of new antifungal therapeutics.

### Natural and anthropogenic selection pressures in co-evolution

Natural selection and co-evolution shape fungal defense traits through ecological interactions such as microbial competition, nutrient limitation, and host immune pressure. At the same time, human activities impose strong anthropogenic selection pressures, particularly through the use of antimicrobial agents in medicine and agriculture. Inappropriate or excessive use of antifungal agents can favor the selection of variants with higher tolerance by fostering increased drug efflux capacity or favoring the survival of strains with altered drug targets, allowing these phenotypes to increase in abundance when selection is sustained (Fisher et al. 2022). In a clinical context, *C. auris* and *C. glabrata* may acquire resistance via target-site changes and enhanced efflux activity, whereas *A. fumigatus* frequently develops triazole resistance through mutations in *CYP51A* (Lee et al. 2023). Similar selective dynamics also occur outside the clinic. Reports of shared resistance-associated *CYP51A* variants in environmental and clinical *A. fumigatus* populations emphasize the need to view resistance emergence across human, agricultural, and environmental settings and to align surveillance and stewardship accordingly.

Host–pathogen relationships can be understood as a co-evolutionary arms race in which fungal traits that promote persistence are countered by host defenses, and vice versa. Co-evolution between early land plants and fungal symbionts illustrates the long history of such interactions in terrestrial colonization and the evolution of compatibility factors (Benucci et al. 2020). Likewise, the crayfish pathogen *Aphanomyces astaci* has been associated with the evolution of specialized immune features in different crayfish species, with genomic studies revealing lineage-specific adaptations (Boštjančić et al. 2022).

Long-term association with humans has also shaped fungal strategies and host immunity. *C. albicans*, a common commensal yeast, can evade immune responses through hyphal transformation and secretion of immune-interacting molecules such as Pra1 (Zipfel et al. 2011), as well as secretion of aspartic proteases that affect host proteins (Gropp et al. 2009). In turn, host recognition pathways involving receptors such as Dectin-1 and cytokine networks, including IL-17/Th17 responses, play a central role in antifungal defense (Gaffen et al. 2011). Population-level evidence supports an extended association between *C. albicans* and humans, consistent with long-term adaptation to host environments (Lott et al. 2005).

Fungi also co-evolve with bacteria within complex communities, producing outcomes ranging from mutualism to antagonism, with consequences for ecosystem and agricultural function (Steenhoudt and Vanderleyden 2000; Deveau et al. 2018). Within these communities, HGT and horizontal chromosome transfer (HCT) can accelerate fungal adaptation. For example, transfer of *ToxA* has been implicated in the emergence of new fungal strains affecting wheat and in altered plant–pathogen responses (Mehrabi et al. 2011). More broadly, microbiomes serve as reservoirs of genetic diversity and selectable traits, and studies of plant-associated communities highlight the contributions of both bacteria and fungi to plant health and disease outcomes (Mubeen et al. 2021).

### Horizontal gene transfer (HGT)

HGT of the *ToxA* gene is a definitive example of how mobile genetic elements foster the emergence of virulence in fungal plant pathogens. *ToxA* encodes a host-selective necrotrophic effector that interacts with the wheat susceptibility gene *Tsn1*, triggering programmed cell death and facilitating infection. This effector-triggered susceptibility converts host defense into a vulnerability, thereby enhancing fungal pathogenicity (Mehrabi et al. 2011). The *ToxA* gene has been horizontally transferred to *Parastagonospora nodorum*, *Pyrenophora tritici-repentis*, and *Bipolaris sorokiniana* via a 14-kb transposon, *ToxhAT*, which includes both coding and regulatory sequences. This transposon carries terminal inverted repeats characteristic of a type II DNA transposon, suggesting that transposition was the primary mechanism of transfer (McDonald et al. 2019).

Recent studies have shown that the *ToxhAT* transposon is embedded within larger mobile elements known as “Starship transposons” (Frontier, Sanctuary, and Horizon), which can capture and mobilize the *ToxA* gene from different fungal genomes (Liu et al. 2025). These mobile genomic islands function not only as vehicles for HGT but also as hotspots of structural genome evolution, contributing to the rapid adaptation of pathogens to new hosts (Gourlie et al. 2022). HGT can also involve the transfer of both core and lineage-specific chromosomes, as observed in *Fusarium oxysporum*, thereby increasing genomic plasticity and adaptive versatility (Vlaardingerbroek et al. 2016). Importantly, HGT is not confined to plant pathogens. In clinical settings, horizontal transfer of resistance plasmids and transposons has been documented in fungi such as *Candida* spp. and *A. fumigatus*, facilitating the rapid dissemination of triazole resistance across species and environments (Morogovsky et al. 2022). These findings reveal a shared evolutionary adaptive mechanism between agricultural and clinical fungi, in which mobile genetic elements act as bridges connecting resistance landscapes across domains.

The acquisition of bacterial genes through HGT has significantly enhanced the metabolic and adaptive capacity of fungal pathogens. For example, *Fusarium verticillioides* has acquired bacterial genes that improve its ability to adapt to fluctuating environments and enhance pathogenicity (Gao et al. 2019). Gene clusters obtained through HGT also play a critical role in sporulation and pathogenicity in *A. alternata* (Wang et al. 2019). The ability to acquire genes from unrelated organisms allows fungi to expand the boundaries of conventional genetic inheritance and rapidly obtain novel traits that enhance infection potential and environmental adaptability. Notably, HGT represents a key mechanism for the acquisition of new resistance and virulence traits.

Recent studies have further highlighted the importance of HGT in fungal pathogens across both clinical and natural environments. Investigations of the fungal microbiome in cystic fibrosis patients demonstrate that HGT contributes to pathogen genetic diversity and adaptability, supporting survival within the host environment (Kim et al. 2015). Inter-species transfer of resistance plasmids in clinical settings further emphasizes the role of HGT in microbial evolution, including in fungi (Kocer et al. 2020). Although HGT was once considered rare in eukaryotes, accumulating evidence indicates that it is more prevalent and impactful in fungi than previously recognized. In this regard, more than 90 gene transfer events have been documented between species of *Magnaporthales* and *Colletotrichum*, primarily enhancing plant cell wall degradation, a key determinant of fungal pathogenicity (Qiu et al. 2016).

### Effect of HGT on fungal pathogenicity and drug resistance

**Figure 3.**
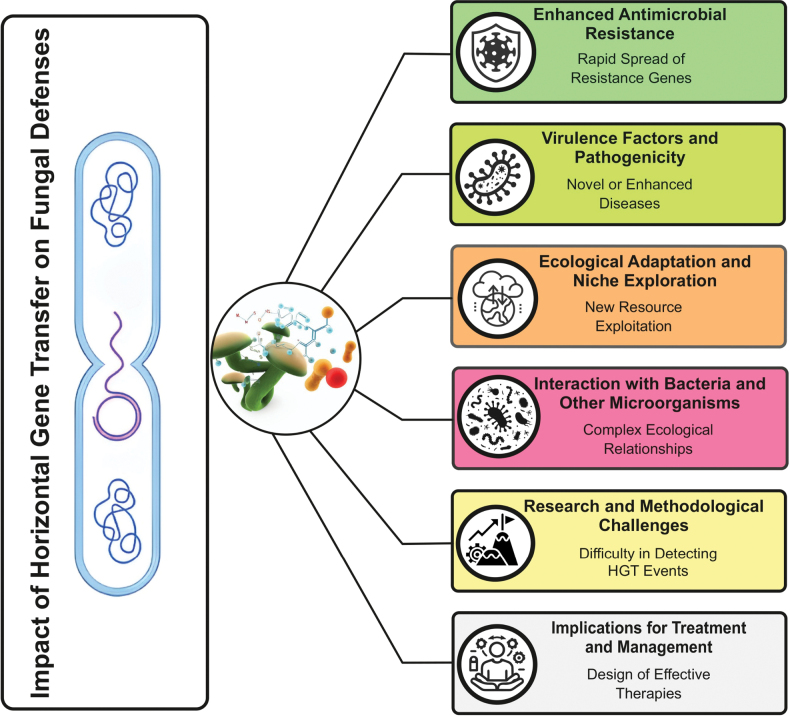
The diverse effects of horizontal gene transfer (HGT) on the defensive strategies of fungi.

HGT contributes not only to fungal adaptive capacity but also directly enhances fungal pathogenicity. For example, the rice blast fungus *M. oryzae* produces a diverse array of effectors, including members of the MAX (Magnaporthe Avrs and *ToxB*-like) effector family, which are critical for disease progression and virulence (Le Naour-Vernet et al. 2023). Novel virulence factors, such as vanadium chloroperoxidase (*MoVcpo*), have also been identified as key contributors to pathogenicity in *M. oryzae* (Nie et al. 2022). These effectors and virulence factors intensify the dynamic interaction between pathogen virulence and host defense, promoting the emergence of increasingly virulent fungal strains (Gupta et al. 2021). Fig. 3 illustrates the substantial impact of HGT on fungal defenses, highlighting both its biological consequences and associated challenges.

HGT enables the rapid transfer, dissemination, and stabilization of genes responsible for AMR, thereby contributing to the increasing prevalence of antifungal resistance in fungal populations. It also facilitates the acquisition of virulence factors, which can lead to the emergence of novel or more severe fungal diseases. In addition, HGT allows fungi to adapt to new ecological niches and exploit resources more effectively, enhancing survival under variable environmental conditions. Gene exchange between fungi and other microorganisms also plays a crucial role in shaping complex ecological interactions. Despite its importance, detection of HGT events remains challenging, and HGT-driven resistance and pathogenicity complicate the development of effective antifungal therapies and the control of fungal infections.

As described above, HGT enables fungi to expand ecological niches and strengthen defense responses. For example, transfer of the *ToxA* gene from *Stagonospora nodorum* to *Pyrenophora tritici-repentis* broadened the host range of the latter to include wheat (Mehrabi et al. 2011). Similarly, acquisition of genes that enhance plant cell wall degradation and neutralization of host defenses, as observed in *Phytophthora ramorum*, underscores the importance of genetic exchange in fungal pathogenicity (Richards et al. 2011). Inter-kingdom HGT between fungi and bacteria, such as *Pseudomonas* species, has enhanced fungal nutrient processing, metabolic capacity, and pathogenic traits. Transfer of plant expansion genes to fungal pathogens has facilitated more effective interactions with plant hosts (Nikolaidis et al. 2014). Fungal pathogens have also acquired genes that enable them to overcome plant resistance mechanisms and expand host range (Li et al. 2018; Wang and Ruan 2020).

**Figure 4.**
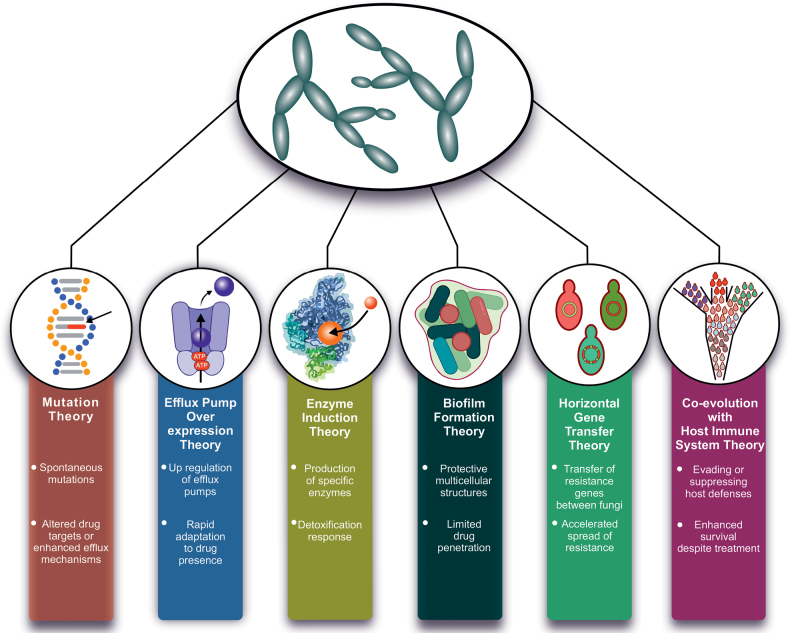
Hypotheses regarding the fundamental mechanisms that contribute to the emergence of fungal resistance against antifungal agents.

Fig. 4 presents six non-mutually exclusive theories explaining the emergence of antifungal resistance: the Mutation Theory, Efflux Pump Overexpression Theory, Enzyme Induction Theory, Biofilm Formation Theory, HGT Theory, and Co-evolution with Host Immune System Theory. This framework categorizes these drivers as contributing to either innate or acquired resistance, providing a structured overview of a multifaceted problem. The Mutation Theory proposes that spontaneous genetic mutations altering drug targets or enhancing efflux mechanisms drive acquired resistance. The Efflux Pump Overexpression Theory suggests that fungi upregulate efflux systems to expel antifungal agents, reducing intracellular drug concentrations; this process may represent an intrinsic trait in some species and an acquired response in others. The Enzyme Induction Theory emphasizes production of enzymes that detoxify antifungal compounds, thereby supporting survival. The Biofilm Formation Theory argues that biofilms act as protective barriers limiting drug penetration, enabling persistence despite antifungal exposure, and may contribute to both innate and acquired resistance. The HGT Theory highlights the transfer of resistance genes among fungi, facilitating rapid dissemination of acquired resistance. Finally, the Co-evolution with the Host Immune System Theory explains how fungi evolve mechanisms to evade or suppress host immunity, enhancing survival even during antifungal therapy. Collectively, these processes illustrate the diverse pathways through which fungi develop innate and acquired resistance, presenting significant challenges for effective treatment and long-term management.

### Hybridization

Hybridization can simultaneously amplify both defense-related traits (e.g., thermotolerance, stress adaptation) and specific AMR traits (e.g., drug efflux and target modification), illustrating how evolutionary processes can blur the distinction between general adaptability and targeted resistance. Hybridization in fungal species can significantly enhance the virulence and adaptability of both human and agricultural fungal pathogens. Hybridization in species such as *C. neoformans* and *Cryptococcus deneoformans* provides genomic plasticity, enabling these fungi to rapidly adapt to environmental stresses and acquire resistance to antifungal drugs. This enhanced adaptability is often fostered by aneuploidy and loss of heterozygosity, which allow hybrids to exhibit greater fitness and survival compared with their parental strains (Samarasinghe et al. 2020). The mitochondrial genome in *Heterobasidion annosum*, a hybrid fungal pathogen, plays a critical role in regulating virulence (Olson and Stenlid 2001). Hybridization in human pathogens such as *Candida metapsilosis* has also led to the development of both innate and acquired virulence traits, including changes in drug sensitivity and thermotolerance, which enhance the ability of the pathogen to infect human hosts (Del Olmo et al. 2025). As observed in plant pathogens, hybridization in clinical fungi can similarly result in increased pathogenicity and drug resistance. For example, hybridization events in *C. neoformans* × *C. deneoformans* have generated lineages with enhanced environmental survival and clinical persistence, mirroring adaptive outcomes seen in hybrid plant pathogens such as *Heterobasidion irregulare* × *H. annosum* (Giordano et al. 2018; Samarasinghe et al. 2020). Hybridization thus creates novel genetic backgrounds that facilitate the rapid acquisition of resistance.

Hybridization among fungal species strongly influences pathogenicity, resistance profiles, and overall adaptability, affecting both natural ecosystems and agricultural systems. In *H. irregulare* and *H. annosum*, hybridization has been shown to alter mitonuclear interactions, with the mitochondrial genome playing a central role in determining virulence and saprophytic growth. Interactions between nuclear and mitochondrial genes ultimately shape the fitness and pathogenicity of hybrid lineages (Giordano et al. 2018). More broadly, advances in genomics have provided insight into the genetic basis of virulence and drug resistance in fungal pathogens, revealing how specific mutations contribute to increased pathogenicity and acquired resistance (Schikora-Tamarit and Gabaldón 2022).

Hybridization also extends to interactions involving hybrid plants and fungal pathogens. Hybrid plants can exhibit altered resistance dynamics that, in turn, influence the virulence and evolutionary trajectories of their associated fungal pathogens, reshaping both plant defense mechanisms and pathogen evolution (Carlsson-Granér et al. 1999). In crop systems, hybridization events in fungal pathogens have led to the emergence of more virulent strains. This phenomenon is particularly evident in mildew pathogens affecting wheat and rye, where hybridization has produced strains capable of overcoming host resistance, such as the mildew pathogen infecting triticale (Müller et al. 2021). These hybridization events generate novel fungal lineages with distinct virulence profiles, increasing outbreak risk and posing substantial challenges for crop protection (Yuzon et al. 2023).

Although hybridization in fungal pathogens can promote more aggressive disease phenotypes, it can also contribute to disease resistance in crops. For example, hybridization events in plant-pathogenic fungi, such as *Ustilago maydis* and *Sporisorium reilianum*, have resulted in altered virulence and infection patterns. These hybrid pathogens often display novel interactions with their plant hosts, influencing disease progression and host defense responses (Storfie and Saville 2021). In addition, interspecific hybridization in fungi such as *Saccharomyces* has produced broad phenotypic diversity in responses to antifungal drugs. Quantitative trait locus mapping has identified hybrid-specific genomic regions associated with resistance to fluconazole, micafungin, and flucytosine (Visinoni et al. 2024). Moreover, hybridization in plant-pathogenic fungi affecting cereals has been linked to enhanced fitness and adaptation to changing host conditions, driving rapid evolutionary responses under selective pressures such as host resistance and chemical treatments (Henningsen et al. 2024). Similarly, hybridization events in human pathogens, including species of *Candida*, have been associated with the emergence of more virulent lineages with improved host adaptation and increased resistance to antifungal drugs (Mixão and Gabaldón 2018).

### One Health: an integrated framework of fungal AMR

The One Health framework is based on the premise that human, animal, and environmental health are interconnected and that fungal AMR emerges from interactions across these domains. Resistance evolution in fungi is driven not only by clinical antifungal exposure but also by extensive agricultural fungicide use, environmental contamination, and ecological selection pressures that operate across ecosystem and host boundaries (Meis et al. 2016). In human health settings, azole-resistant *A. fumigatus* infections are associated with high mortality, particularly among immunocompromised patients. Pan-azole resistance linked to the TR34/L98H alteration in the *cyp51A* gene has been widely reported in clinical isolates and is associated with treatment failure and limited therapeutic options (Denning and Bowyer 2013; Meis et al. 2016).

Animal and environmental domains play a major role in generating and maintaining resistant fungal populations. Agricultural application of triazole fungicides, which are chemically similar to clinical azoles, imposes strong selection pressure in soils, compost, and crop-associated environments. Consistent with this, resistant *A. fumigatus* has been isolated from farms, air, compost, and hospital-adjacent environments, indicating environmental persistence and potential occupational exposure (Gonçalves et al. 2020; Burks et al. 2021). Soil and plant microbiomes can act as reservoirs for resistant strains, while residual azoles may sustain long-term selection. Resistant spores can then disperse via wind, water, trade, and human activity, enabling regional and global spread (Wang et al. 2018).

These domains are linked by multiple pathways that facilitate the movement of resistance traits and resistant propagules within the One Health framework. Proposed routes include HGT that disseminates resistance determinants such as *cyp51A* variants, widespread dispersal of resistant spores transporting TR34/L98H lineages over long distances, and the shared use of azole compounds in agriculture and medicine that promotes cross-resistance (Chowdhary et al. 2013). Collectively, these interacting pressures make *A. fumigatus* a valuable model organism for studying the origins and spread of fungal AMR within the One Health framework.

The TR34/L98H resistance mechanism in *cyp51A*, characterized by a 34-bp tandem repeat in the promoter region and a leucine-to-histidine substitution, exemplifies a One Health pathway in which environmental selection and clinical impact converge. First detected in both environmental and clinical isolates in the Netherlands, this genotype has since been reported across multiple regions, including parts of Asia, Africa, and the Americas (Chowdhary et al. 2013; Rocchi et al. 2021). Genetic analyses have identified closely related TR34/L98H genotypes in environmental and hospital-associated isolates, supporting a link between fungicide-exposed environments and clinical disease. Resistant spores have also been detected in hospital air, agricultural soils, and patient samples, indicating ongoing ecological cycling across settings (Gonzalez-Jimenez et al. 2021).

## Conflicting evolutionary theories

The evolution of fungal defense mechanisms remains one of the most debated topics in mycology, based on diverse lines of evidence, including comparative genomics, phylogenetics, population genetics, and ecology, which support different interpretations. Disagreements focus on both mechanism and tempo. Mechanistically, one position argues that defense traits are shaped primarily by vertical inheritance through gradual, lineage-specific diversification, including gene duplication and divergence. Comparative genomic analyses suggest that many defense-associated systems, such as stress-response pathways, cell wall biosynthesis genes, and efflux transporters, are conserved across major fungal clades, supporting early origins followed by later modification rather than frequent acquisition from external sources (Moran et al. 2011). Large-scale genomic comparisons further indicate that many novel gene families arose endogenously during major evolutionary transitions, consistent with the view that internal innovation has shaped core fungal traits (Wu et al. 2022).

In contrast, another position emphasizes horizontal processes as drivers of rapid innovation. HGT has been documented across fungal lineages and can introduce genes linked to pathogenicity or resistance. For example, plant pathogens in *Magnaporthales* and *Colletotrichum* have been reported to share extensive sets of horizontally acquired genes involved in plant cell wall degradation, traits that can directly enhance host exploitation (Qiu et al. 2016). Similarly, the chytrid pathogen *Batrachochytrium dendrobatidis* has been shown to acquire bacterial-derived genes associated with detoxification or antibiotic resistance, supporting the argument that HGT contributed to its emergence as a highly virulent pathogen (Sun et al. 2016). Beyond HGT, interspecific hybridization can also generate abrupt genomic change by combining large portions of genomes into mosaic or polyploid lineages. The existence of hybrid lineages in *Candida* and powdery mildew fungi has been associated with altered virulence and niche expansion (Steensels et al. 2021). Collectively, these findings complicate strictly tree-like evolutionary models by highlighting reticulate histories in which vertical inheritance, HGT, and hybridization operate jointly rather than as mutually exclusive processes.

Debate also persists over tempo, specifically whether resistance traits represent ancient capacities or recent responses to human activity. Some researchers argue that many resistance-associated mechanisms, including efflux systems and general stress-response regulation, predate modern antifungal use and represent standing variation that can be selected under new conditions (Wisecaver et al. 2014). Others contend that intensive exposure to antifungal agents, particularly widespread azole use in medicine and agriculture, has accelerated resistance evolution through selective sweeps, local adaptation, and gene flow between environmental and clinical populations (Gladieux et al. 2014). Integrative perspectives reconcile these positions by proposing a pluralistic model in which gene duplication and vertical inheritance provide a stable evolutionary backbone, while HGT and hybridization generate episodic bursts of novelty during ecological transitions. In this view, combined phylogenetic, population, and ecological analyses are essential for explaining how fungi balance long-term genetic conservation with rapid innovation (Romeijn et al. in press). Suppl. material 1: fig. S2 summarizes controversies and debates in evolutionary theories of fungal defense.

### Potential biases and limitations in evolutionary studies

The study of fungal evolutionary processes involves several limitations and biases that can strongly influence how results are interpreted and generalized, particularly when distinguishing the evolutionary origins of innate versus acquired resistance (Table 2). Suppl. material 1: fig. S3 summarizes these challenges and highlights how methodological, ecological, and conceptual constraints complicate efforts to explain when, where, and why resistance traits arise. Overall, current evolutionary models are shaped not only by biological reality but also by uneven data coverage and practical constraints on sampling and experimentation.

**Table 2.** Challenges in the study of fungal evolution, especially regarding fungal defense mechanisms.

Challenge	Issue	Impact	Reference
Sampling bias	Selection focus	Skewed understanding	Panzeri et al. 2008
Technological limitations	Technological constraints	Detection limitations	Loffler and Boutellier 2009
Phylogenetic uncertainties	Tree construction	Misinterpretations	Rangel et al. 2015
Ecological context	Lab vs. real-world	Limited applicability	Bronfenbrenner 1986
Economic and geographic biases	Focus on economic/geographic importance	Neglect of diversity	Fowler and Fox 2009; Bromham et al. 2016
Ethical considerations	Ethical dilemmas	Research limitations	Hasan et al. 2021
Interdisciplinary barriers	Cross-disciplinary communication	Holistic understanding barriers	Pischke et al. 2017

A major issue is sampling and taxonomic bias. Research has disproportionately focused on economically or clinically important genera such as *Candida*, *Aspergillus*, and *Cryptococcus*, which can overemphasize patterns of acquired resistance while underrepresenting the broader diversity of fungal strategies. Early-diverging and environmentally dominant lineages are often missed because of primer choice and sampling design in DNA-based community surveys, and commonly used ITS and LSU metabarcoding workflows do not reliably capture these groups, resulting in incomplete phylogenetic coverage (Reynolds et al. 2022). Large-scale monitoring efforts similarly indicate that standard soil and air sampling approaches can overlook geographically restricted taxa or fungi with low spore production, biasing evolutionary inference toward widespread and easily detected species (Ovaskainen et al. 2024).

Technological and methodological constraints further shape evolutionary conclusions. PCR-based amplification and sequencing can distort inferred community composition and gene presence, because primer selection, barcode region choice, and sequencing depth can influence diversity estimates as strongly as true environmental differences (Tedersoo et al. 2015). Detection efficiency is also affected by amplicon length and GC content, which can systematically undercount rare taxa and early-diverging clades (Seeber et al. 2022). Even within well-studied groups, limited marker resolution across ITS, LSU, or SSU datasets can produce inconsistent taxonomic assignments, complicating downstream evolutionary reconstruction (Monod et al. 2022).

Analytical bias and phylogenetic uncertainty introduce additional limitations. Alignment quality, tree-building approaches, and model selection can generate artefactual relationships, while marker-dependent signal variation can distort evolutionary distance estimates, as shown in genera where commonly used loci differ substantially in discriminatory power (Rampersad et al. 2014). In genome-scale analyses, incomplete or inconsistent annotation further affects inference about key processes such as HGT and selection, meaning that apparent evolutionary patterns may partly reflect annotation gaps rather than underlying biology (Hugaboom et al. 2023).

Insufficient consideration of ecological context represents another recurring shortcoming. Many evolutionary studies rely on laboratory conditions that simplify or exclude natural selective pressures, producing models that may not accurately reflect fungal adaptation in natural ecosystems (Anderson and Cairney 2004). Factors such as competition, fluctuating resources, and microbial co-evolution can be central drivers of resistance in nature, yet are often absent from experimental designs. Without integrating environmental data, interpretations may become heavily gene-focused while remaining limited in their ability to explain the ecological conditions under which resistance traits are maintained or favored (Naranjo-Ortiz and Gabaldón 2019).

### Real-world impact of fungal resistance to antimicrobial agents

**Figure 5.**
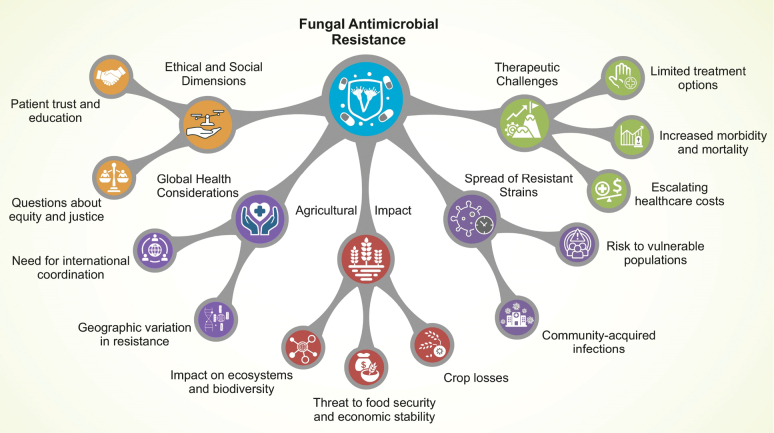
The clinical consequences and practical effects of fungal antimicrobial resistance.

Fungal resistance to antimicrobial compounds is an escalating global problem that affects both human health and agricultural production. This section examines the clinical and real-world consequences of antifungal resistance, with the major pathways of impact and outcomes summarized in Fig. 5. Overall, fungal resistance to antimicrobial agents represents a rapidly intensifying crisis within the One Health framework, as it threatens human health, food security, and ecosystem stability worldwide. Importantly, resistance to antifungal agents has now been reported across all major classes of antifungal compounds used in medicine and agriculture (Fisher et al. 2022).

Antifungal resistance in clinical settings has increasingly contributed to treatment failure in invasive infections, particularly among immunocompromised patients. The global burden of fungal disease is substantial, with estimates exceeding 150 million infections annually, which in recent years has contributed to millions of deaths (Mudenda 2024). Reflecting this threat, the World Health Organization has designated *C. auris* and *A. fumigatus* as critical priority pathogens because of their multidrug-resistant profiles and high associated mortality (Ramos et al. 2025). Resistant infections frequently lead to longer hospital stays, higher healthcare costs, and increased mortality, with particularly severe outcomes reported for invasive candidiasis in many settings.

The rise of azole-resistant strains of *A. fumigatus* illustrates how clinical and environmental reservoirs are becoming increasingly interconnected. Pan-azole-resistant strains are now reported across continents and are being detected even in patients without prior azole exposure, suggesting acquisition from environmental sources rather than exclusively from in-host evolution (Burks et al. 2021). Well-characterized resistance mechanisms, including *cyp51A* alterations such as TR34/L98H and TR46/Y121F/T289A, can confer broad resistance to medical azoles and sharply constrain therapeutic options (Gonçalves 2017). These trends highlight the growing difficulty of managing infections when first-line antifungals become ineffective and alternative drugs are limited or more toxic.

Beyond healthcare, antifungal resistance also threatens agricultural sustainability and food security. Plant diseases caused by fungi and oomycetes are estimated to destroy a substantial fraction of global crop yields each year, undermining economic stability, nutrition, and food security (Fisher et al. 2020). Azole fungicides remain essential for protecting major crops, yet they share molecular targets with medical azoles, creating the potential for cross-resistance between agricultural and clinical contexts (Pintye et al. 2024). Resistant pathogens and resistant lineages associated with genera such as *Fusarium*, *Candida*, and *Aspergillus* have been discussed in the context of movement across soil, crops, built environments, and clinical settings, complicating prevention and control strategies (Williams et al. 2024). Agricultural intensification can further strengthen environmental selection, including through repeated azole exposure and practices that introduce additional antimicrobial residues into fields, although the strength of direct genetic links between agricultural azole use and specific clinical resistance outcomes varies by region and study (Barber et al. 2020; Corrêa-Junior et al. 2024).

The economic and societal costs of antifungal resistance are therefore substantial. Rising healthcare expenditures, productivity losses, and reductions in agricultural yield can cumulatively impose large national and global burdens, and projections suggest that long-term costs could reach very high levels if resistance continues to expand and treatment options become increasingly limited (Inoue and Minghui 2017). These impacts are often greatest in low- and middle-income countries, where access to rapid diagnostics and second-line antifungals is limited, raising concerns about equity in both exposure to risk and capacity to respond (Woods et al. 2023).

Given these cross-sectoral drivers and outcomes, policy responses increasingly emphasize One Health coordination. Integrated strategies that combine clinical stewardship, reform of agricultural practices, environmental monitoring, and coordinated surveillance are widely viewed as essential for slowing resistance emergence and reducing its spread (Picot et al. 2022). Current recommendations also highlight the value of internationally harmonized antifungal policies, global resistance databases such as AFRbase, and more sustainable fungicide use to reduce the selective pressures that foster resistance across interconnected systems (Jain et al. 2023).

### Detection and monitoring

Detecting and monitoring fungal resistance using both traditional and advanced techniques is essential for effective resistance management and the selection of appropriate therapeutic agents. Advanced molecular methods, such as digital PCR (dPCR), have substantially improved monitoring by providing sensitive, quantitative, and rapid detection of fungicide resistance at the genetic level. Notably, dPCR has proven effective in detecting low-frequency alleles and resistance-associated mutations (Zulak et al. 2018). Other advanced approaches, including next-generation sequencing (NGS) and PCR-based assays, have further enhanced the ability to identify and characterize resistance genes in clinical samples. These molecular tools are particularly valuable in the management of invasive fungal diseases (IFDs), where early and accurate diagnosis can significantly influence patient outcomes. Molecular techniques have also addressed many limitations of traditional culture-based methods, which are often slow and may fail to identify cryptic species (Kidd et al. 2020).

The integration of site-directed genetic manipulation using CRISPR technologies and artificial intelligence (AI) is providing new approaches in medical mycology for understanding and combating fungal infections. One notable development is the creation of a CRISPR activation (CRISPRa) platform in *C. albicans*, which enables targeted overexpression of genes involved in fungal pathogenesis and drug resistance. This platform provides a real-time approach for studying resistance mechanisms and elucidating how resistance develops (Gervais et al. 2022). CRISPR–Cas9 is increasingly applied in medical mycology to investigate and potentially overcome antifungal resistance. Future applications, including systems that promote rapid spread of engineered traits through populations, programmable base editors, and additional DNA- and RNA-based technologies, have the potential to transform diagnostics and therapeutic interventions (Morio et al. 2020).

CRISPR and RNA interference (RNAi) technologies also offer promising tools for addressing antifungal-resistant strains in regions such as Africa, where surveillance systems for azole resistance in *A. fumigatus* are limited. These approaches are important for improving the accuracy and efficiency of fungal pathogen surveillance, which is central to controlling resistant strains (Achilonu et al. 2024). CRISPR–Cas13 systems are emerging as powerful diagnostic tools, enabling rapid and accurate detection of resistant fungal strains and offering potential improvements in infection control strategies (Ortiz-Cartagena et al. 2023).

Artificial intelligence (AI) is increasingly applied to the detection and prediction of AMR. AI-based tools are being used to analyze resistance patterns, identify biofilm-forming organisms, and predict emerging resistance trends (Mishra et al. 2024). Integrating genomics, CRISPR technologies, and AI is expected to accelerate resistance detection and facilitate identification of novel resistance mechanisms (Olatunji et al. 2024). As AI-driven approaches continue to develop, they are likely to substantially enhance diagnostics and control of drug-resistant fungal pathogens (Mairi et al. 2025). Advanced detection tools are equally relevant in agricultural and environmental monitoring. For example, CRISPR-based assays developed for clinical *Aspergillus* resistance could be adapted to track azole-resistant strains in soil and crop microbiomes, enabling a One Health approach to resistance surveillance that integrates clinical, agricultural, and environmental health.

### Management and control

As the global problem of antifungal resistance continues to escalate, managing and overcoming fungal resistance will require a comprehensive, multifaceted approach. Effective AMR management involves responsible use of antifungal agents across sectors, including medicine and agriculture, to prevent the emergence and spread of resistant fungal strains. A review by Hart et al. (2019) examined antifungal stewardship (AFS) interventions in the United States, assessing their impact on clinical outcomes such as mortality, hospital length of stay, appropriate antifungal selection, and time to therapeutic intervention. The authors found that, although AFS interventions improved appropriate antifungal selection and reduced overall antifungal consumption, they did not significantly affect mortality or hospital stay duration. These findings highlight the complexity of addressing fungal resistance and underscore the need for a holistic approach that includes robust stewardship programs.

Combination therapy represents another strategy for managing fungal resistance. The use of multiple antifungal agents with different mechanisms of action can counteract resistance, enhance treatment efficacy, and reduce the likelihood of pathogens developing acquired resistance. Prevention and education are also pivotal components of resistance management. Increasing public awareness of responsible antifungal use and the risks associated with overuse and misuse of antimicrobial agents is essential, as is emphasizing adherence to prescribed treatments. Integrated AMR management requires collaboration among researchers, clinicians, policymakers, and other stakeholders (Osset-Trénor et al. 2023).

## Clinical practices and real-world impact

Fungal resistance to antimicrobial agents is a pressing real-world challenge with profound therapeutic consequences. Its clinical impact is broad and multifaceted, influencing treatment decisions, patient outcomes, and global health security (Suppl. material 1: table SS1, Fig. 5). Because the resistance strategies discussed above enable fungi to persist despite drug exposure (Table 3), addressing this challenge requires coordinated action that links clinical practice with public policy, agriculture, waste management, and ethical governance. Clinical management has increasingly shifted toward structured, evidence-driven approaches, particularly through antifungal stewardship (AFS), diagnostics integration, and multidisciplinary coordination. Antifungal stewardship programs aim to optimize antifungal use by standardizing protocols, improving diagnostic certainty, and monitoring prescribing patterns (Hamdy et al. 2017). Structured AFS initiatives in hospital settings have demonstrated substantial reductions in antifungal consumption and improvements in prescription appropriateness without increasing mortality or resistance outcomes. Multidisciplinary stewardship teams have also reduced antifungal expenditures while maintaining clinical effectiveness (Whitney et al. 2019; Zhang et al. 2024). In addition to stewardship, diagnostics-driven care is essential for minimizing unnecessary therapy and enabling early, targeted treatment. Rapid molecular and antigen-based tools, including β-D-glucan and galactomannan assays, MALDI-TOF, and PCR, can shorten time to pathogen identification and support de-escalation, thereby reducing overall antifungal exposure. However, limited access and slow turnaround times remain major barriers in low-resource settings (Chakrabarti et al. 2022; Schelenz et al. 2025). Therapeutic drug monitoring (TDM) and antifungal susceptibility testing are also increasingly incorporated into clinical workflows to individualize dosing, improve efficacy, and reduce toxicity, particularly in patients with variable pharmacokinetics or high risk of treatment failure (Gupta et al. 2023).

**Table 3.** Key strategies utilized by fungal pathogens to evade host immune defenses.

Strategy	Target	Effect	Reference
Masking PAMPs	β-glucans, PRRs	Avoid immune detection	Childers et al. 2020
Immunosuppressive molecules	Complement system, regulatory T cells	Create favorable survival environment	Marcos et al. 2016
Biofilm formation	Phagocytosis, antifungal drugs	Protect the community from immune attack	Hernández-Chávez et al. 2017
Modulating cell death	Apoptotic pathways	Allow more time to spread	Chai et al. 2009
Antigenic variation	Surface antigens	Evade adaptive immune response	Luo et al. 2013

Importantly, real-world drivers of resistance extend far beyond hospital settings. Widespread antimicrobial use in animal and crop production can intensify environmental selection and contribute to the emergence of resistant strains. For example, antimicrobial use to treat animals in Africa was estimated to be in the thousands of tons between 2015 and 2019, with even greater usage reported in many high-income regions, underscoring the need for judicious regulation given ecological spillover risks (Mshana et al. 2021). Environmental contamination is also influenced by waste-management practices, as improper disposal and inadequate wastewater treatment can disseminate antifungal residues and resistant organisms, reinforcing selection outside clinical environments. These pressures are compounded by the increasing vulnerability of human populations, as growing numbers of immunocompromised individuals face severe fungal infections. At the same time, the pipeline for developing new classes of antifungal compounds remains limited, highlighting the need for sustained investment in research and therapeutic innovation (Denning 2022; Vitiello et al. 2023).

Surveillance serves as the critical link between stewardship and diagnostics-driven care by tracking emerging resistance and guiding intervention strategies. Regional and global monitoring efforts have highlighted threats such as azole-resistant *A. fumigatus* and multidrug-resistant *C. auris*, including evidence linking resistance patterns to environmental fungicide exposure and hospital transmission. These findings reinforce the need for coordinated One Health action across sectors (Arastehfar et al. 2020). In practice, multidisciplinary teams are increasingly recognized as best practice for managing invasive fungal infections, with improvements in guideline adherence and patient outcomes reported when infectious disease physicians, pharmacists, microbiologists, and other specialists collaborate on timely diagnosis and optimized therapy pathways (Soni et al. 2024).

Finally, ethical and equity considerations are central to any durable response. Ensuring equitable access to diagnostics and effective antifungal therapies, maintaining transparency and accountability in policy, and reducing inappropriate antifungal use in agriculture and prophylactic settings are essential to preserving antifungal efficacy and protecting vulnerable populations (Rabaan et al. 2023). Innovative strategies that reduce antimicrobial pressure in food systems and the environment, together with continued development of new therapeutics and implementation capacity, will be critical for limiting the clinical and societal burden of antifungal resistance (Au et al. 2021).

## Interdisciplinary approaches

### Future challenges

Combating the growing problem of fungal pathogens resistant to antimicrobial agents is a complex challenge that requires coordinated progress in therapeutic development, mechanistic research, environmental stewardship, and clinical management. Resistance mechanisms discussed earlier in this review, including target mutations, efflux activity, and biofilm formation, have reduced the efficacy of current treatments (Geddes-McAlister and Shapiro 2019). However, advances in fungal genetics and pathogenesis continue to identify promising targets and strategies, including combination regimens and exploration of non-traditional sources such as medicinal plants, which may expand available therapeutic options (McCarthy et al. 2017; Marquez and Quave 2020). Because antifungal resistance is increasingly shaped by interconnected clinical and environmental pressures, application of a One Health framework linking human, animal, and ecosystem health will be central to future mitigation efforts (Fisher et al. 2022).

A primary challenge is the limited arsenal of systemic antifungal agents and the slow pace of drug discovery. Only a small number of drug classes are widely available for systemic infections, and resistance continues to erode efficacy through efflux activation, target modification, and biofilm-associated tolerance (Sanglard 2016; Vitiello et al. 2023). Future progress will depend on the development of agents with novel mechanisms and targets, supported by approaches such as synthetic biology and emerging material-based strategies designed to improve specificity and reduce host toxicity. However, high costs, regulatory barriers, and slow translation from laboratory to clinic remain major bottlenecks (Khalifa et al. 2024).

Diagnostic innovation is equally critical, as early and accurate identification enables targeted therapy and reduces unnecessary exposure to antifungal agents. Although next-generation molecular assays and metagenomic approaches offer faster detection and resistance profiling, major implementation gaps persist, particularly in low-resource settings where cost, infrastructure, and standardization limit routine use. Closing these gaps is essential for diagnostics-driven stewardship to function effectively and to improve clinical outcomes globally (Pham et al. 2024).

Environmental and agricultural reservoirs represent another critical frontier. Extensive azole fungicide application can favor selection of resistant strains, including *A. fumigatus*, and there is evidence that these lineages contribute to human infection, blurring the boundary between environmental selection and clinical disease (Williams et al. 2024). Risk reduction will require stewardship and regulation that limit unnecessary non-medical azole use, along with monitoring of soils, crops, and other human-associated environments to detect resistance emergence early and prevent amplification (Hollomon 2015; Pintye et al. 2024).

Finally, progress will depend on the establishment of stronger global surveillance systems and coordinated policy responses. The WHO’s 2022 list of fungal priority pathogens has helped prioritize threats, but surveillance capacity remains uneven across regions, and many countries lack the laboratory infrastructure and trained personnel required for routine susceptibility testing and reporting (Smith et al. 2023; Arendrup et al. 2024). Future challenges are also being shaped by climate change, which may expand fungal geographic ranges and favor thermotolerant, drug-resistant species such as *C. auris*, increasing the need for integrated monitoring and prevention across economic and public health sectors (Lockhart et al. 2023; Fisher et al. 2024). Renewed interest in natural products and plant-derived compounds may also provide potential multi-target scaffolds for antifungal development. However, standardization, safety evaluation, and clinical validation remain significant hurdles before natural products can be widely adopted (Argüelles et al. 2024; Long and Li 2024).

### Ethical considerations

Efforts to combat fungal resistance face not only scientific challenges but also ethical and policy constraints. Limited antifungal therapeutic options, global disparities in drug access, and the absence of rapid diagnostic assays exacerbate mortality risks associated with resistant species such as *Candidozyma auris* (formerly *Candida auris*), *Candida* spp., and *Aspergillus* (Vitiello et al. 2023). The widespread use of antifungal agents in agriculture further complicates the balance between crop protection and long-term human and environmental health (Marquez and Quave 2020). Governmental policies are required that incentivize drug innovation, promote stewardship through education and surveillance, and regulate agricultural fungicide use. Global cooperation is also essential to harmonize standards and ensure equitable access to treatments, while ethical frameworks must guide research, safeguard communities, and prevent misuse of scientific findings (Pandey et al. 2018).

### Integration of genetics, biology, pharmacology, and other disciplines

Addressing fungal resistance requires an interdisciplinary approach that moves beyond broad endorsements of collaboration to implement actionable, cross-disciplinary frameworks. Concrete examples demonstrate both the power and necessity of this approach. Agricultural azole fungicide use has been directly linked to the emergence of clinically resistant *A. fumigatus* strains, particularly those carrying the TR34/L98H mutation in the *cyp51A* gene. Cross-sectoral studies have detected identical resistant genotypes in both farm environments and clinical infections, confirming environmental selection as a key driver. For example, the TR34/L98H mutation has been identified in both lung samples from patients and soil from fungicide-treated fields, establishing a direct clinical–agricultural connection (Rocchi et al. 2021). Similar patterns have been reported globally, including in China (Chen et al. 2020), Portugal (Gonçalves et al. 2020), and the United States (Hurst et al. 2017). At a global scale, population genetic studies have shown that environmentally driven resistance alleles, such as TR34/L98H and TR46/Y121F/T289A, have spread internationally through selective sweeps, defined as rapid increases in the frequency of advantageous resistance alleles initiated by agricultural fungicide pressure (Sewell et al. 2019; Bottery et al. 2024).

Technological innovations, particularly gene-editing tools such as CRISPR, offer promising avenues for developing novel therapies that enable direct genetic modification of fungi or host organisms. These technologies are expected to contribute substantially to advances in personalized medicine and precision treatment strategies (Murugaiyan et al. 2022). Recent studies have also identified new potential drug targets, including fungal fatty acid synthesis pathways, that have demonstrated efficacy against drug-resistant yeast species. Ultimately, interdisciplinary collaboration, combined with careful consideration of ethical, environmental, and societal factors, will form the foundation for responsible and effective antifungal strategies. A holistic approach is essential to ensure that scientific advances translate into treatments that are both effective and equitable, while safeguarding public health and the environment.

To systematically foster such synergy, we propose the establishment of One Health Fungal Resistance Consortia. This coordinated framework would unify clinical, veterinary, agricultural, and environmental efforts by creating shared biorepositories and interoperable genomic databases that are isolate-specific and include harmonized metadata across sectors to accelerate surveillance and discovery. These consortia would also catalyze cross-domain diagnostics by supporting collaborations in which hospital laboratorians and plant pathologists jointly validate resistance-detection platforms, including CRISPR-based assays, against markers applicable to both healthcare and agricultural contexts. Crucially, they would enable joint evolutionary risk assessment through interdisciplinary teams of evolutionary biologists, epidemiologists, and agricultural pathologists who could develop models to predict how fungicide deployment in specific crops may influence regional resistance patterns over defined horizons, such as five years, including potential downstream impacts on nearby healthcare facilities.

Interdisciplinary collaborations have already advanced understanding of fungal resistance and generated innovative solutions with transformative potential. Accordingly, efforts should be expanded to promote international collaboration and scientific exchange. Key areas for collaborative research include interdisciplinary studies of fungal pathogens, application of gene-editing technologies, discovery of plant-derived secondary metabolites with antifungal activity, identification of signaling pathways, development of fungal molecular databases, and advances in nanomaterials (Tang et al. 2022). Strengthening international partnerships among chemists, biologists, pharmacologists, and other researchers will further accelerate antifungal drug discovery.

Integration of microbiology, genetics, bioinformatics, and global surveillance networks can enhance the capacity to identify, monitor, and manage resistant strains. Collaborations among scientists, ecologists, and policymakers are fostering more sustainable agricultural practices, while joint efforts in health economics and policy analysis are improving clinical solutions in healthcare systems. Engagement among healthcare providers, educators, and community leaders has also strengthened public awareness and antimicrobial stewardship. Ethical challenges are being addressed through cooperation among scientists, ethicists, and regulatory bodies. Collectively, these collaborations underscore the importance of breaking down disciplinary barriers and developing comprehensive strategies to confront the rise of fungal resistance to therapeutic agents. For example, research on efflux pump inhibitors in *C. albicans* has informed similar investigations in plant-pathogenic fungi, while genomic insights from the rice blast fungus (*M. oryzae*) have guided virulence studies in human pathogens. Such cross-domain application of research findings accelerates discovery and supports the development of antifungal strategies that are effective in both clinical and agricultural settings.

### Challenges in interdisciplinary research

Interdisciplinary research on fungal resistance faces several challenges, including the complexity of fungal genomes, which frequently undergo insertions, deletions, rearrangements, and gain-of-function mutations, making interpretation of genetic changes difficult. Animal models used in clinical research present another limitation, as they may fail to predict clinical outcomes. Studies typically rely on young, otherwise healthy animals under controlled conditions that do not reflect the genetic diversity or comorbidities of human patients (Kontoyiannis 2017). Imprecise terminology, such as references to “sustainability” or “innovation,” can also lead to miscommunication, as can biases that prioritize specific methodologies or data types. Institutional factors, including insufficient funding and systems that undervalue interdisciplinary work, further constrain progress. Intellectual property disputes, challenges in methodological integration, and lack of data standardization can also hinder interdisciplinary collaboration. Ethical and cultural considerations, including alignment of principles across diverse cultural contexts and respect for local practices, must likewise be addressed. Despite these barriers, the benefits of interdisciplinary collaboration in addressing fungal resistance outweigh the challenges. Targeted efforts, including expanded training opportunities, supportive institutional policies, open communication, and sustained attention to ethical considerations, can strengthen collaborative success. These measures may also serve as a model for interdisciplinary solutions to other scientific and societal challenges.

## Recommendations and limitations

### Recommendations for future research, policy, and practice

This review of fungal defenses, AMR, and their evolution yields several key recommendations for future research, policy, and clinical practice. Research efforts should expand interdisciplinary collaborations aimed at filling knowledge gaps and applying innovative methodologies. Genomics and artificial intelligence should be integrated within a global framework that respects cultural diversity. Policy measures should strengthen regulatory frameworks, promote international coordination, support public awareness initiatives, and enhance real-time assessment of emerging fungal resistance. Clinical and practical strategies should emphasize personalized medicine informed by genetic data, integration of advanced technologies, sustainable agricultural practices, and partnerships with community organizations. Collectively, these recommendations represent a multifaceted approach grounded in innovation, collaboration, responsible governance, and community engagement. By recognizing the biological, environmental, and cultural complexity of fungal resistance, this integrated strategy seeks to harness the combined strengths of science, policy, technology, ethics, and society to address the global challenge of fungal resistance to antimicrobial therapeutics.

### Limitations of the present review

Although this review provides a comprehensive discussion of fungal defense mechanisms, AMR, and their evolution, several limitations should be acknowledged. These include incomplete coverage of certain subfields, potential selection bias in source material, and reliance on specific methodologies that may oversimplify an interdisciplinary synthesis. The discussion may also reflect Western perspectives, potentially underrepresenting cultural diversity and regional sensitivities. In addition, some recent advances may not be included because of the rapid pace of technological and scientific development, and projections of long-term impacts should be regarded as suggestive rather than definitive. Ethical considerations, including potential conflicts of interest and sensitivity to ethical dilemmas, also warrant attention. These limitations do not diminish the value of this review. Rather, they underscore the need for continued critical reflection and debate. Above all, this review emphasizes the importance of ongoing research, methodological rigor, cultural inclusivity, and ethical integrity in addressing the complex and evolving challenge of fungal resistance to antimicrobial agents.

## Conclusion

This review provides an overview and discussion of the interconnected dynamics of fungal defense mechanisms, AMR, and their evolution within a One Health framework. We argue that fungal defense and AMR are shaped by shared evolutionary pressures across clinical, agricultural, and environmental domains rather than arising in isolation, and we present evidence to support this view. Basal fungal defenses, including cell wall integrity pathways, efflux systems, enzymatic detoxification, and adaptive regulatory networks, provide the foundation from which clinically and agriculturally relevant resistance can emerge under intensified selection. In parallel, HGT, hybridization, and human-driven selection enable resistance traits to cross species boundaries and move between ecosystems. Documented links between agricultural azole use and resistant *A. fumigatus*, the spread of virulence and resistance determinants via mobile genetic elements, and adaptive outcomes associated with hybridization reinforce the conclusion that resistance is best understood as an ecosystem-level phenomenon. Recognizing this continuum, however, is only a starting point, because compartmentalized research and fragmented management approaches are poorly suited to addressing a global challenge. Progress against fungal AMR requires a shift from reactive, domain-specific responses to proactive, integrated strategies. These include interdisciplinary coordination through shared frameworks and resources, evolutionary risk forecasting to anticipate cross-domain consequences of interventions, development of dual-purpose approaches applicable to both human and plant pathogens, and coordinated, equitable surveillance systems that track resistance across One Health interfaces. Because resistant fungal pathogens succeed through the coupling of defense, adaptation, and resistance across environments, effective containment must be equally integrated, linking scientific innovation with coordinated governance to ensure long-term protection of public health, food systems, and ecosystem integrity.

## Additional information

### Use of AI

No use of AI was reported.

### Author ORCIDs

Yuan Sui https://orcid.org/0000-0002-3034-0642

Mir Muhammad Nizamani https://orcid.org/0000-0002-8709-9212

Entaj Tarafder https://orcid.org/0000-0002-3680-3433

Yong Wang https://orcid.org/0000-0003-3831-2117

### Data availability

All of the data that support the findings of this study are available in the main text or Supplementary Information.

## Supplementary materials

Supplementary material 1 Supplementary information Data type docx https://binary.pensoft.net/file/1521364This dataset is made available under the Open Database License (http://opendatacommons.org/licenses/odbl/1.0/). The Open Database License (ODbL) is a license agreement intended to allow users to freely share, modify, and use this Dataset while maintaining this same freedom for others, provided that the original source and author(s) are credited. Yuan Sui, Mir Muhammad Nizamani, Entaj Tarafder, Hai-Li Zhang, Qian Zhang, Krishnendu Acharya, Jit Sarkar, Ghulam Muhae-Ud-Din, Yong Wang
