# Supplementary material for: Interconnected worlds: a comprehensive review of fungal defenses, antimicrobial resistance, and their evolutionary dynamics
Source: IMA Fungus. 2026 Jan 27;17:e171995. doi: 10.3897/imafungus.17.171995 (PMC13034067; doi:10.3897/imafungus.17.171995)
Supplement: Supplementary material 1 — Supplementary information [file imafungus-17-e171995-s001.docx]

**Supplementary figures**


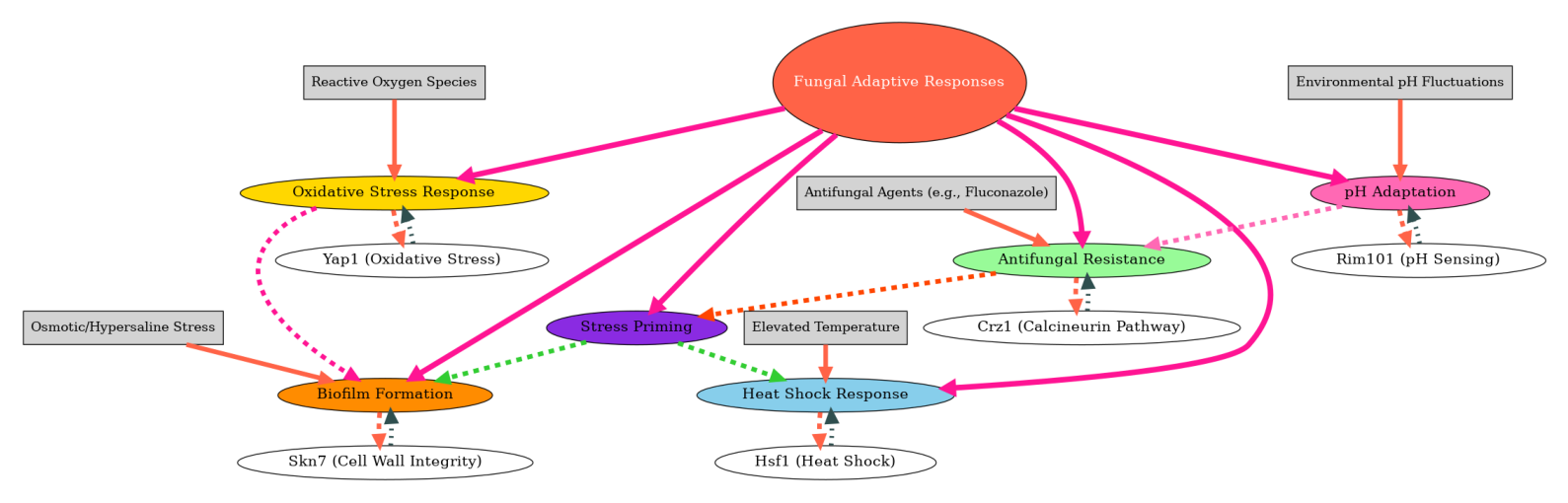


**Figure S1.** Fungal adaptive responses to environmental stressors.


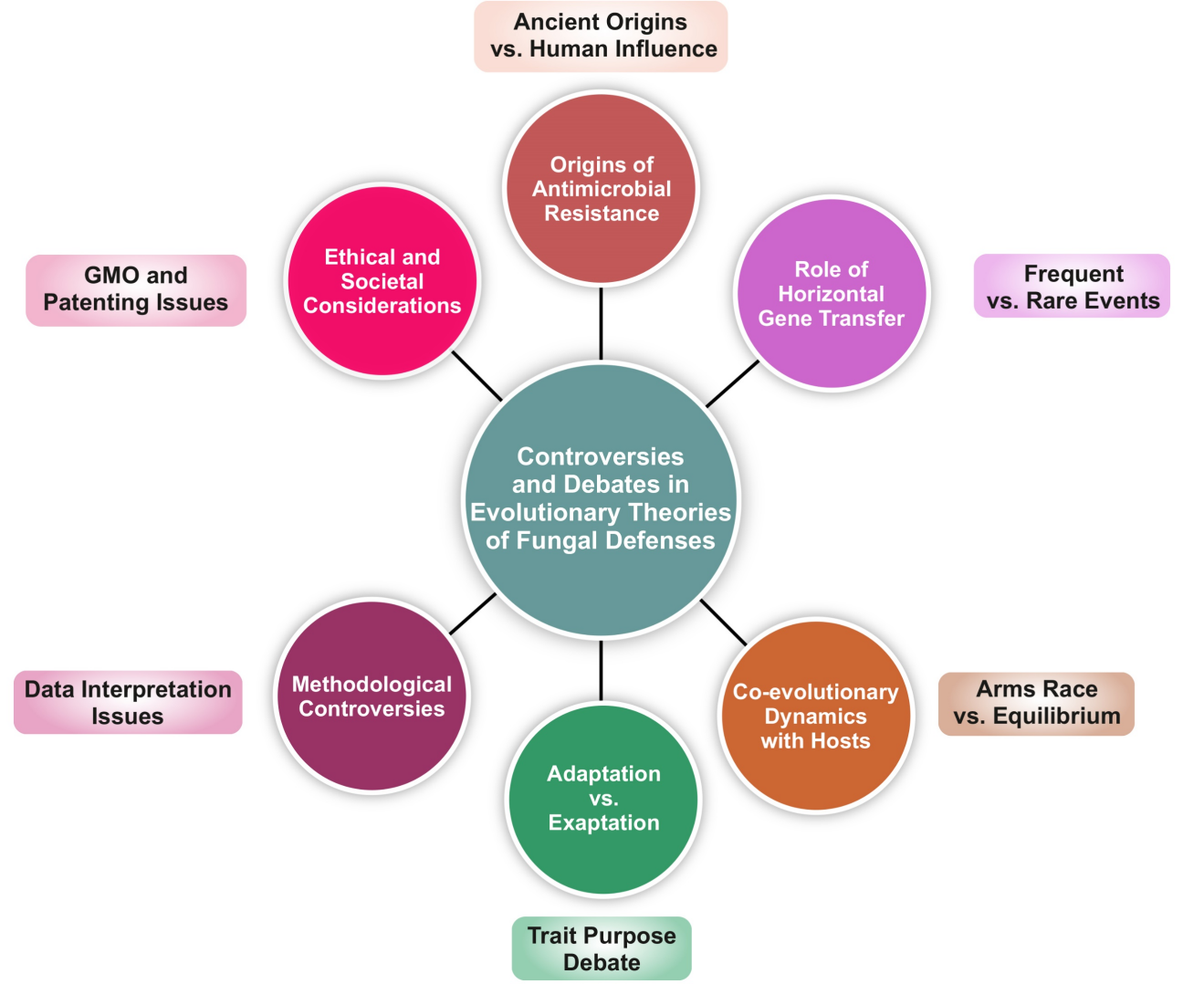


**Figure S2.** Fundamental theories and debates surrounding the development of defensive strategies in fungi.


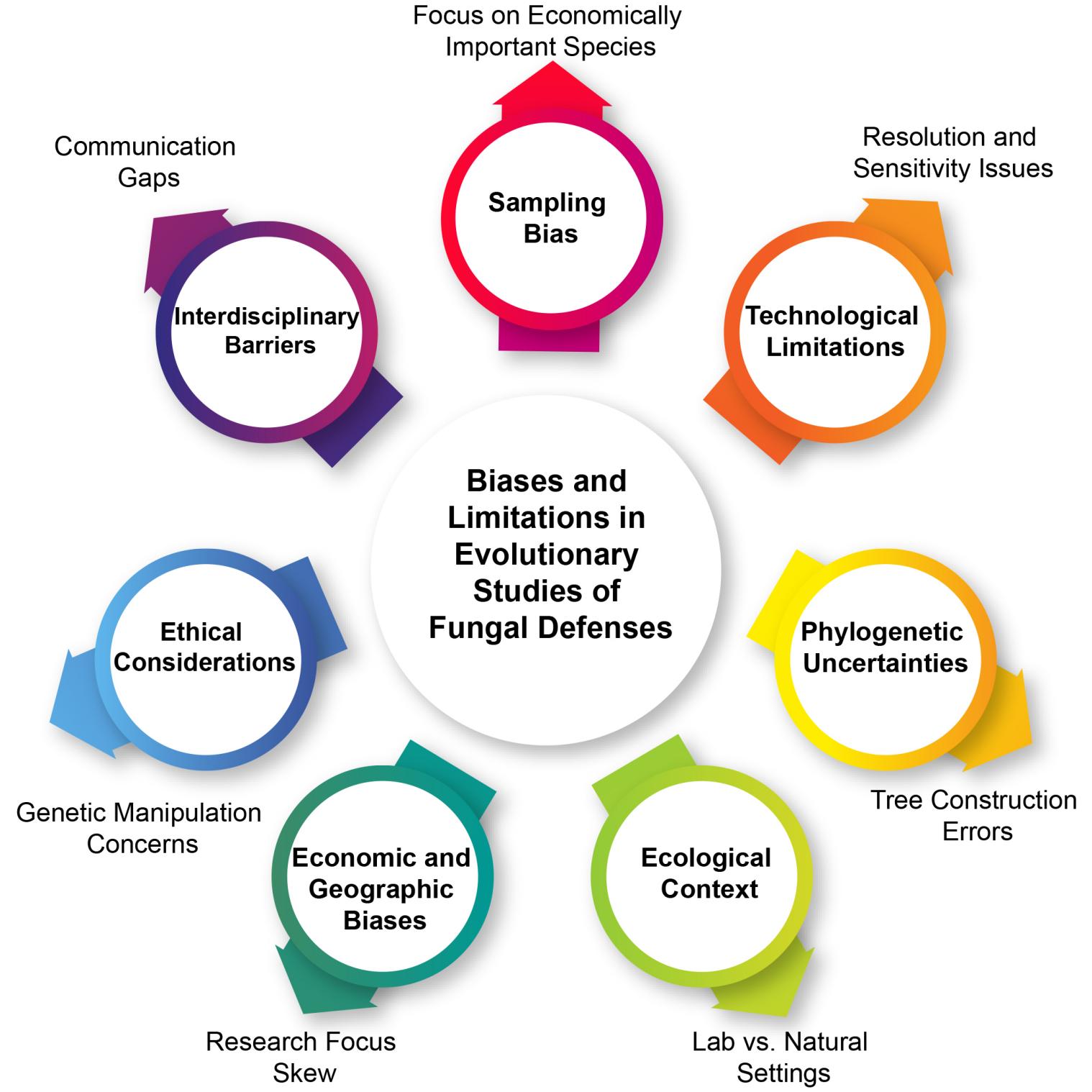


**Figure S3.** Possible biases and constraints in research on the development of fungal defense strategies.

**Table S1.** Challenges and real-world impact of antifungal resistance.

| Challenges | Description | Impact | Location | Reference |
| --- | --- | --- | --- | --- |
| Therapeutic challenges | Resistance limits treatment options, raising healthcare costs. | Clinical, economic | Healthcare facilities | McCarthy et al. 2017 |
| Spread of resistant strains | Resistant strains spread, endangering patients and communities. | Public health | Healthcare and community | Martins-Santana et al. 2023 |
| Agricultural impact | Crop losses threaten food security and affect ecosystems. | Agricultural, environmental | Farms and ecosystems | Fones et al. 2020 |
| Global health considerations | Global concerns require international coordination. | Global health | Worldwide | Martins-Santana et al. 2023 |
| Ethical and social dimensions | Limited access raises ethical questions and erodes trust. | Ethical, social | Healthcare and society | Arastehfar et al. 2020 |
